# Collective almost synchronization-based model to extract and predict features of EEG signals

**DOI:** 10.1038/s41598-020-73346-z

**Published:** 2020-10-01

**Authors:** Phuong Thi Mai Nguyen, Yoshikatsu Hayashi, Murilo Da Silva Baptista, Toshiyuki Kondo

**Affiliations:** 1 Department of Computer and Information Sciences, Tokyo University of Agriculture and Technology, Tokyo, 184-8588 Japan; 2 Biomedical Science/Engineering, School of Biological Sciences, University of Reading, Reading, RG6 6UR UK; 3 Institute for Complex System and Mathematical Biology, University of Aberdeen, Aberdeen, AB24 3UE UK

**Keywords:** Biophysical models, Computational models, Computational neuroscience

## Abstract

Understanding the brain is important in the fields of science, medicine, and engineering. A promising approach to better understand the brain is through computing models. These models were adjusted to reproduce data collected from the brain. One of the most commonly used types of data in neuroscience comes from electroencephalography (EEG), which records the tiny voltages generated when neurons in the brain are activated. In this study, we propose a model based on complex networks of weakly connected dynamical systems (Hindmarsh–Rose neurons or Kuramoto oscillators), set to operate in a dynamic regime recognized as Collective Almost Synchronization (CAS). Our model not only successfully reproduces EEG data from both healthy and epileptic EEG signals, but it also predicts EEG features, the Hurst exponent, and the power spectrum. The proposed model is able to forecast EEG signals 5.76 s in the future. The average forecasting error was 9.22%. The random Kuramoto model produced the outstanding result for forecasting seizure EEG with an error of 11.21%.

## Introduction

One of the fundamental questions in neuroscience is about how individual neuronal activity represented by the action potential combines to generate the observed collective dynamics at the population level, which can be recorded by electroencephalography (EEG) measurements. At the macroscopic level, the neuronal networks of the cortex can be modeled as a spatially continuous network^1,2^ that reproduces this same collective behavior but with a specified spatial resolution. Understanding how the function at the cellular level translates into the function at the macroscopic level is clouded by an experimental limitation that impedes one from measuring the neuronal activities of thousands, millions, or even billions of neurons^3^. Thus, to fill this gap between the micro and the macro states, mathematical modeling at the cellular level plays a critical role in describing neuronal activities^4–7^.

EEG measurement is a powerful noninvasive tool for measuring brain macroscopic collective activity, with higher temporal resolution, and has been widely used to understand the electrophysiology of brain activity correlated with not only motor coordination but also cognitive functions. EEG signals are complex, and they have stochastic, nonlinear, and nonstationary characteristics, which makes the application of classical methods of time-series analysis controversial^8,9^.

Fourier analysis (to obtain the power spectrum or to decompose the signal into its frequency components) is one such classical method that has been studied extensively in the literature^10^. Other classical model-based (parametric) methods include the auto-regressive (AR), moving average (MA), auto-regressive moving average (ARMA), and auto-regressive fractional integrated moving average (ARFIMA)^11–13^. These methods focus on the analysis of the signal in the time domain. Recently, there has been a surge, owing to interest in machine learning methods, in attention in modeling EEG signals using neuronal models^14^.

Fourier analysis has been widely applied to the treatment of EEG signals, which has led to the discovery of the so-called event-related desynchronization/synchronization^15,16^. These changes in intensity at a certain frequency range are known to be neuronal correlates of motor intention and cognitive functions^17,18^.

At the heart of Fourier analysis lies the concept of the Fourier series based on the assumption that a function that satisfies general Dirichlet conditions can be represented as a sum of trigonometric functions. Therefore, a signal in the time domain can be decomposed into its frequency modes, and as the number of modes increases, the quality of the signal that can be reconstructed improves. Nonlinear and nonstationary signals can also satisfy Dirichlet’s conditions; thus, it is plausible, at least from the mathematical perspective, to study EEG signals with Fourier decomposition. However, there has been a continuous discussion whether decomposing strong nonstationary signals such as EEG signals is plausible^19^. Moreover, clearly, the EEG should not be created out of the sum of the never-changing periodic signals that oscillate with such a static distribution of Fourier frequency modes.

Our work considers that EEG signals can be modelled as a sum of the action potentials spanned by oscillators that model the action potentials of the biological neural cells. Therefore, to better understand the dynamical nature of EEG signals, we use a neuronal model-based approach.

Recent work has shown that EEG signals can be optimally modeled by a complex network of chaotic Hindmarsh–Rose (HR) neurons that are weakly connected and behaving in the so-called collective almost synchronization (CAS) state^20^. This modeling approach suggested that brain activities can be generated by weakly interacting chaotic neurons. Therefore, it proposes the idea that EEG signals can be successfully modeled or decomposed by a basis of chaotic signals generated by neurons that are weakly connected in a complex network.

In this study, we show that the optimal method to reconstruct EEG signals from a complex network of oscillators is constructing an output function (a weighted average signal of the action potential of several neurons) that only considers orthogonal signals. Using this approach, we show that the orthogonal set of time series generated by complex networks of both HR and Kuramoto oscillators minimizes the error function to fit the EEG data under several conditions.

HR neural networks operating in the CAS regime have been previously shown to effective for modeling EEG signals^20^. However, in this study, we show that the discrepancy between the real and estimated EEG signals in the model could be reduced substantially if the EEG model is not constructed from time series collected from randomly selected neurons of the neuron network, but by using a compressed set of independent vectors produced by the principal component analysis (PCA) from the entire series of data generated by the network. We also show that to model EEG data, one does not need to rely on networks of neuronal models, but can also use networks formed by coupled Kuramoto phase oscillators. In addition, our results corroborate those in Ref.^20^ by showing that the best models to predict the EEG signals are obtained when the oscillators are weakly connected, satisfying the conditions for the existence of the CAS regime.

We show that our model can reproduce and predict both healthy and epileptic EEG signals, as well as the characteristics of the EEG signals, Hurst exponent, and power spectrum of experimental EEG signals.

## Related work

Various models of neuronal activity that can be used to understand and reproduce EEG signals exist. Some studies have considered a stochastic limit cycle oscillator to model EEG signals^21–23^. They can also be modeled using networks of stochastic coupled nonlinear oscillators, with the dynamic unit described by the Duffing oscillator^24^ or by Jansen’s single-column model^25^. It was shown that a stochastic Duffing–van der Pol oscillator network model could capture the key characteristics of EEG signals, such as its time-varying power spectrum, Shannon entropy, and sample entropy of healthy controls and patients with a brain disorder. Recently, the phenomenon of CAS was introduced to model how spatial and temporal patterns can appear from complex networks in which neurons interact with each other with small coupling strengths^26^. The CAS phenomenon emerges when a certain number of individual neurons experience an approximately constant local mean field from other connected neurons. In the work of Ref.^20^, it was shown that networks of HR neurons could produce data that would best fit EEG signals when the network was set to operate in the CAS regime.

After a model for the EEG signal is proposed, one should attempt to validate it for future forecasts^27^. EEG signals are known to be high-dimensional, noisy, and difficult to forecast even for short time intervals. However, recent research approaches have shown promising results in the forecasting of these signals, as described in Table 1.

**Table 1:** Related work on the modeling and prediction of EEG signals.

Works	Sampling rate (Hz)	Period of prediction (ms)	Valuation
Autoregression model^11^	500	100	Phase locking value
Autoregression model^12^	64	500	Pearson correlation, mean square error
Artificial neural network and the combination of temporal and frequency of EEG^28^	512	500	Error measurement
Generalized linear model^29^	50	5	Number of spikes count prediction

Our model follows the ideas outlined in the work of Ref.^20^. Similarly, we consider networks of nonlinear (HR neurons and Kuramoto phase oscillators) oscillators weakly connected to operate in the CAS regime. However, our output function to model the EEG uses a time series constructed to be independent of the dynamical units. Moreover, we show that the signals generated by our model reproduce the main features of experimental EEG signals, such as the Fourier spectrum and Hurst exponent.

Using our proposed model, we also aim to understand how fields generated as a sum of action potentials of neurons can predict complex macroscopic signals such as an EEG signal. The first step in our modeling approach is to train an output measurement of the network of nonlinear elements to fit an initial training set of EEG data and, then, to validate the model by finding optimal configurations of the network to best predict a new set of EEG signals (not considered in the training set). Characteristic variables that allow us to change the network configurations to better predict EEG signals are the following: (i) the strength of interactions among the dynamical units forming the networks, (ii) the types of dynamical units (either HR neurons or phase oscillators), and (iii) the topology of the network (random and small-world network). In this study, we considered a set of EEG signals with five different characteristics.

Through a systematic study of different strengths of interactions among the different dynamical units in the networks, we were able to demonstrate the generality of the CAS regime, being independent of the dynamical units. As optimal models of EEG signals were obtained for all network configurations that were set in the CAS regime, we corroborate the idea that CAS is the *de facto* relevant feature to allow a network of dynamical units to be used by a mean field to model EEG signals.

## Results

**Figure 1:**
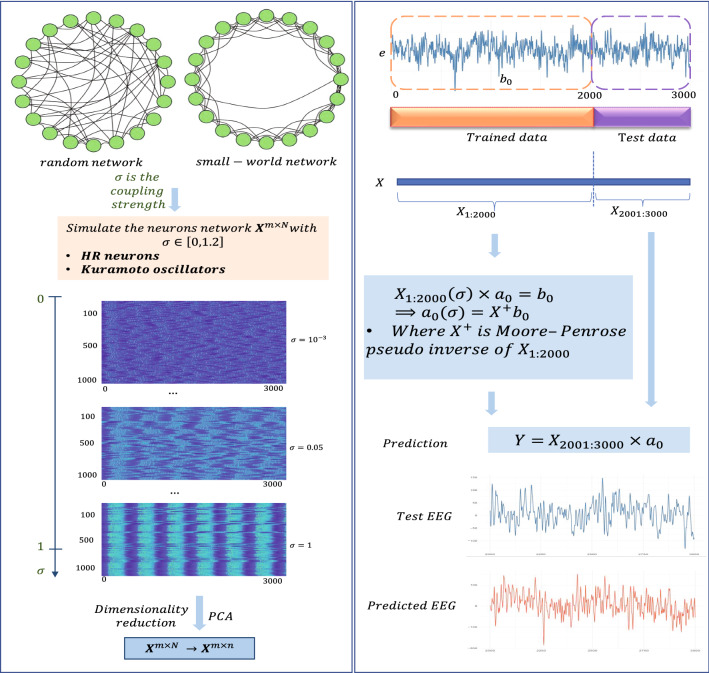
Overview of our method. *Left*: Dynamical networks with two types of topology: random and small-world networks. HR neurons or Kuramoto phase oscillators were used as a dynamical model. The coupling strength was selected to be [0, 1.2]. Data generated by the network were dimension reduced by singular value decomposition (SVD) analysis. *Right*: Datasets were divided into training data and test data. Then, the coupling strengths of the networks that produced the smallest fitting error were selected to generate the matrix *X* from which we calculated the predicted EEG signal (*Y*).

Our model was constructed by considering both EEG signals from healthy subjects and subjects with epileptic conditions. According to a previous work^20^, the output function constructed from an average of weighted (“trained”) time series collected from randomly selected neurons in a network operating in the CAS regime can well reproduce EEG signals. Certain neurons in a network operating in the CAS regime behave as if they are weakly correlated. Therefore, the time series collected from a random selection of neurons in such a network would likely create a set of time series that are roughly independent (“orthogonal”). Our novel approach is to propose a new strategy to select neurons to construct the weighted output function, partially based on this principle. In this study, we used PCA to determine an orthogonal set of neuronal activities as a function of time (see the details in the Methodology section). PCA is a well-known tool that can reduce the dimension of a dataset consisting of variables that are correlated while retaining most of the information^30,31^.

### Summary of methodology

Our model for the EEG was developed on a trained output from an autonomous dynamical complex network, set to operate in the CAS regime. We consider two topologies, random and small-world, and two systems for the node dynamical behavior: HR neurons and phase oscillators. We assume that this network has *N* nodes. The HR networks are described by the system of ordinary differential equations (ODEs) in Eq. (6), and the Kuramoto-like phase oscillator networks are described by Eq. (8). Neurons in the HR network were coupled electrically with a strength given by $\sigma$, and each node in the Kuramoto-like network experiences a coupling strength of ${\tilde{r}}_i$, describing its dynamics coupled to the mean field.

Our networks have $N=1000$ nodes, and we collect a time series from each node with $m=3000$ data points. With these time series, we construct the matrix ${\varvec{X}}^{*} \in {\mathbb {R}}^{m \times N}$, where each column is a time series of length *m* from a node. The dimensionality of this matrix is reduced using singular value decomposition (SVD) to produce a matrix ${\varvec{X}} \in {\mathbb {R}}^{m \times n}$ ($n<N$) with *n* orthogonal columns and still contains 99$\%$ of the total variation of the original matrix, preserving 99$\%$ of all the information of the original matrix. Here, *n* is the number of retained principal components of ${\varvec{X}}^{*}$. This is accomplished using Eq. (21). This percentage of the total information was selected by optimizing the quality of our model.

Using $b_0$ to represent the experimental EEG time series, our network CAS-based EEG model of this experimental signal is denoted by *Y*, which is calculated using Eq. (13), where $Y \in {\mathbb {R}}^{(t_m-t_1) \times 1}$ constructed for the sample interval $[t_1,t_f]$ and $X \in {\mathbb {R}}^{(t_m-t_1) \times n}$ represents the reduced matrix obtained by Eq. (21). $a_0$ represents the vector of the coefficients trained by Eq. (12) and used to produce the output function of the network modeling the EEG signal. The sample interval $[t_1,t_m]$ is an EEG time-series interval [1, 3000], and $[t_1,t_f]$ is the trained interval [1, 2000].

The proposed model was applied to predict the EEG signals of five datasets. The model was evaluated by comparing the experimental EEG signal for the “test data” time windows (the last 1000 data points) with the predicted EEG from the model. The regression model was used to forecasting the EEG, this process is an algebraic one. Each EEG signal is only predicted using its own information. The problem is how to choose the time-interval of the initial or ‘training’ data. Regarding the used data set, 2000 time points for calculating the coefficient is enough to avoid the over-fitting and give the stable results. To further evaluate the performance of our model, we compared the Hurst exponent (related to the long-range correlation) and the power spectrum of the experimental EEG dataset with that obtained from our model, considering four configurations: random HR, small-world HR, random Kuramoto, and small-world Kuramoto models.

Random networks were generated by the Gilbert random graph approach^32^, denoted by G(1000; 0.01), in which every possible edge occurs independently with a probability of $p=0.01$. For the small-world networks, we used the Watts–Strogatz network generation approach^33^ with a rewiring probability equal to $p=0.01$. Both networks have a mean degree of 10. Examples of values for the network characteristics small worldness, average path length, and clustering coefficient for the considered networks are (0.0304, 3.2632, 0.0098) for a random network and (8.0536, 19.3226, 0.6622) for a small-world network, respectively.

Figure 1 depicts a scheme of the methodology adopted in this study. The left panel shows network configurations with snapshot plots of their behavior. The top part shows examples of the two network topologies being considered: random and small-world networks. Each node has dynamics that can be described by either HR neurons or Kuramoto phase oscillators. The networks were simulated with coupling strengths $\sigma \in [0,1.2]$. The bottom shows three snapshots, each for different coupling strengths for networks with $N=1000$ nodes and for 3000 simulated trajectory points. Coherent patterns emerge for higher coupling strengths, and for the purpose of modeling performance, these configurations should be avoided.

The right panel in Fig. 1 represents how we constructed our model and used it to predict EEG data. In the top figures, the dashed boxes mark the time windows considered for the training data, comprising the first 2000 points of the experimental EEG data (*b*), and the testing data, comprising the last 1000 points and that is not used in the training phase of the model. Both the EEG signals and *X* generated by the dynamical networks were split into training data and test data. Prior to the application of the prediction methods, the measured signals of the dynamical networks were reduced by SVD. The upper blue inset box illustrates the calculation of the $a_0$ coefficients (Eq. (12)), and the lower inset blue box shows how we generated the predicted EEG signal using this trained vector of coefficients (Eq. (13)). The bottom two figures show the test data of the EEG and the predicted EEG signal (generated by Eq. (13)).

### Comparison of methods to generate *X*

**Figure 2:**
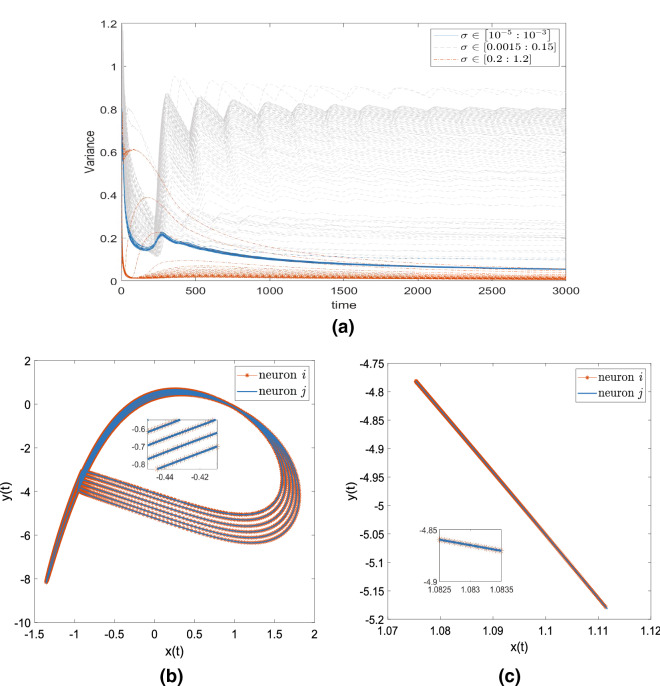
Results validating the two criteria for the HR neurons to exhibit the CAS pattern. (**a**) Variance values of the local mean field of a randomly selected node for all values of coupling strength. (**b**) CAS pattern for two neurons with coupling strength $\sigma =0.001$. (**c**) CAS pattern for two neurons with coupling strength $\sigma =1$.

**Figure 3:**
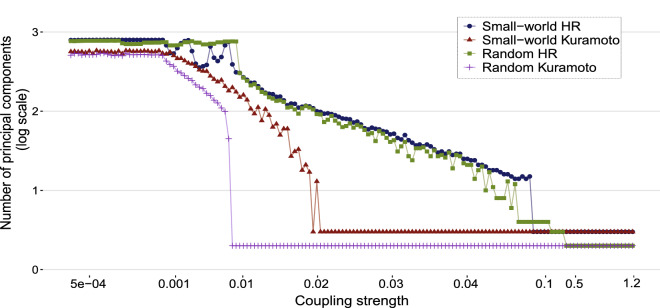
Number of principal components *n* that were retained as a function of the coupling strength $\sigma$ to maintain $99\%$ of the total variance, as in Eq. (20).

The local mean field of the neuron depends on the coupling strength $\sigma$. Therefore, the value of $\sigma$ was tuned from 0 to 1.2 to find the CAS regime. In this regime, we found that $\sigma \le \sigma ^{CAS}$, where $\sigma ^{CAS} \cong 0.001$ for all HR and Kuramoto models. The CAS phenomenon exists when a node has an approximately constant local mean field. If the equation for the CAS pattern presents the coexistence of attractors, nodes are still in a CAS state if the CAS condition is satisfied. Figure 2a shows the variances of the local mean field at every time point. In particular, the variance of the local mean field of neuron *i* at time point *t* is calculated as follows:

1 $$\begin{aligned} Variance_i\left( t \right) =Var\left( \left\{ {\bar{x}}_i \right\} _{t} \right) \end{aligned}$$

where $\left\{ {\bar{x}}_i \right\} _{t}$ is the vector of the local mean field from the starting time point to time point *t*. As time increases, the blue lines show that the variance values of the nodes of the weakly coupled network converge to 0 (**criterion 1**). The variances of the nodes of the strongly coupled network are still high (gray lines). For the coupling strength in [0.2 : 1.2], the variances also converge to 0 (the red lines). However, Fig. 2c shows these neurons are not a stable periodic orbit. In Fig. 2b, the CAS pattern of coupling strength $\sigma =0.001$ described a stable periodic orbit (**criterion 2**).

Our proposed method to generate the matrix *X* is based on reduction using PCA. We considered the matrix ${\varvec{X}}\in {\mathbb {R}}^{m\times N}$ for the entire network simulated considering all $m=1000$ nodes. Then, we reduced it to ${\varvec{X}}\in {\mathbb {R}}^{m\times n}$ using PCA. The new matrices contained the *n* principal components, which were constructed as mixtures of the initial networks. These principal components are uncorrelated.

Understanding how the coupling strength changes behavior in the network and how this affects the ability of PCA is important to reduce the dimension. Figure 3 shows the relation between the coupling strength value and the number of retained principal components for all network models to maintain 99$\%$ of the total variance.

The coupling strengths of networks that produced the smallest fitting error were selected to generate the matrix *X* from which we calculated the predicted EEG signal. These values of $\sigma$ are smaller than 0.001 for both the HR model and the random Kuramoto model.

To justify the novelty of the proposed approach, we compare it with the method proposed in Ref.^20^, in which the nodes of the HR network considered to construct the reduced matrix ${\varvec{X}}\in {\mathbb {R}}^{m\times n}$ are selected randomly.

The value of $\sigma$ was chosen by minimizing the value of the mean absolute error (MAE) in Eq. (14); thus, it is the coupling that creates behavior such that our model fits the best EEG signals. The values of $\sigma$ obtained for all our network models were within the interval determined in which the CAS phenomenon existed, so $\sigma \le \sigma ^{CAS}$.

**Figure 4:**
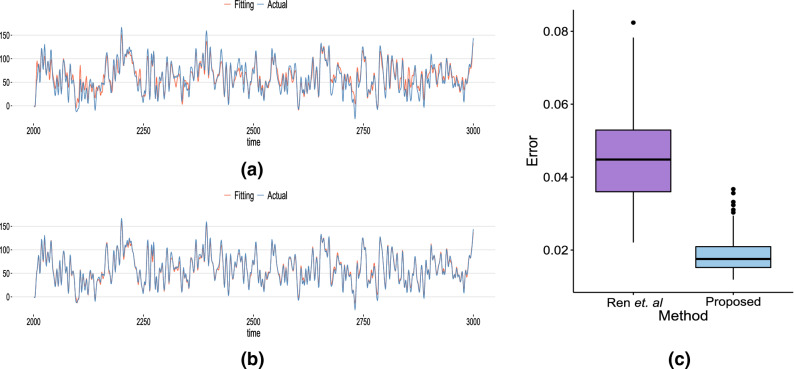
Comparison of the efficiency to model the EEG signals between the method being proposed in this work and that in Ref.^20^ to model EEG signals. (**a**) The red line shows the modeled EEG signal and the blue line shows the actual EEG signal, for the method of^20^. (**b**) The red line shows the modeled EEG signal and the blue line shows the actual EEG signal for our proposed method. (**c**) Error distributions between the EEG signals and modeled curves for the “Ren et al.” method presented in Ref.^20^ (in violet) and the proposed method (in blue). The error analysis was carried out using 100 channels (100 EEG signals) of set A (healthy individuals with closed eyes).

For comparison, the MAEs of the two methods were compared using 100 channels of dataset A (healthy individuals with closed eyes). The results are shown in Fig. 4. Both methods fit the EEG signals well. However, as demonstrated by the distribution of the error in Fig. 4c, our proposed method can fit EEG signals with more than twice the accuracy (Wilcoxon test, two-tailed *p*-value $<0.01$).

### Validation of the two different neuronal models

**Figure 5:**
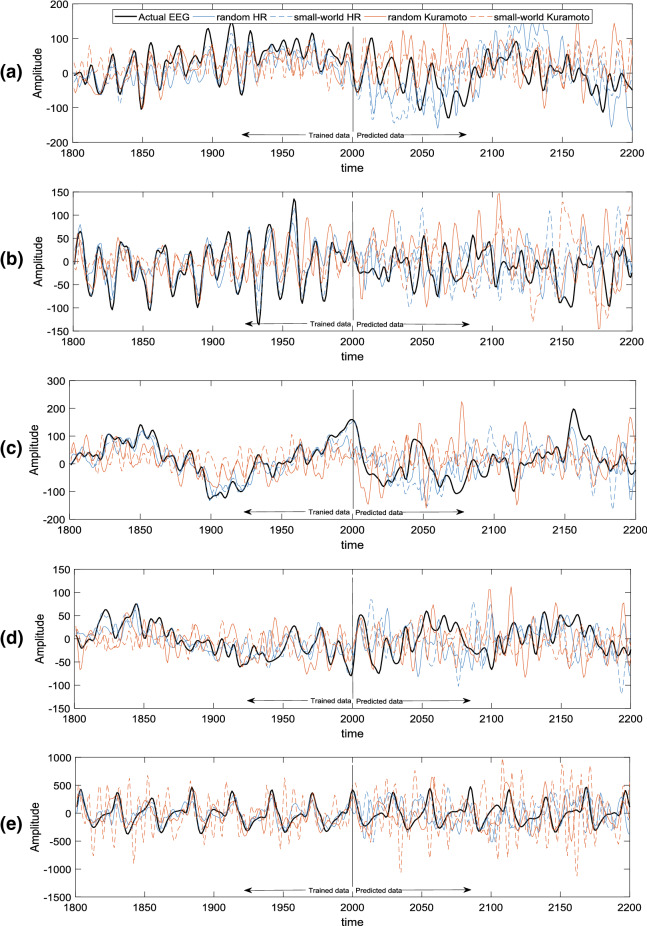
EEG data obtained experimentally, training and prediction of five datasets: A, B, C, D, and E. The black lines show the actual EEG signals. The blue lines show the results from networks of HR neurons, whereas the red lines show the results for the networks of Kuramoto oscillators. The random and small-world network topologies are represented by the solid and dashed lines.

This study used an open-source database from Bonn University^34^: A (closed eyes, healthy records), B (opened eyes, healthy records), C and D (seizure-free interval, epileptic records), and E (during seizure activity, epileptic records). From the five participants for each set, the EEG recordings were obtained using the 10–20 international electrode positioning system. Each set consisted of 100 single-channel EEGs under a sampling rate of 173.61 Hz. The datasets were band-pass filtered (0.5–30 Hz, EEGLAB embedded Fourier infrared (FIR) filter). In this study, 3000 sampling points collected over approximately 17.28 s were used. The first 2000 points were training data and the last 1000 points were predicted data.

A sequence of network configurations was considered, with coupling strength varying within the range of $\sigma \in [0,1.2]$. For a given 2000 sampling points over approximately 11.52 s for a single channel, the weighted parameters of the proposed model were calculated using Eq. (11). Then, MAE values between the EEG signals and the reconstructed EEG signals based on our proposed model approach were calculated using Eq. (14). The predicted signal shown is generated be considering a network whose coupling strength $\sigma$ minimizes the MAE function.

Figure 5 shows several representative single-trial predictions that are used as a typical example from datasets of four models, considering different network configurations, particularly different topologies, with various dynamical units and for a range of values for $\sigma$. These trials demonstrate that random and small-world HR networks (Fig. 5A–C) and the random Kuramoto network (Fig. 5D,E) allow for a predictive signal that can accurately capture the general underlying trend of the data. In particular, the reconstructed EEG signal for the random Kuramoto network can capture prominent peaks in the power spectra (Fig. 5E). The EEG reconstructions that use the small-world Kuramoto networks had the worst modeling performance; that is, they could not capture the general trend and the frequency spectral component of the EEG signal (Fig. 5E).

In addition to conducting an error analysis of our predicted EEG signal, we evaluated our modeling approach by checking whether the generated EEG signals in the predicting regime could reproduce the characteristic features of the power spectrum (comparing similarities with the EEG signals in the frequency domain) and the Hurst exponent (comparing similarities with the EEG signals in the long-term correlations).

#### Error analysis of the predicted EEG signal

The average error scores computed using the MAE quantity in Eq. (14) for the different prediction models are presented in Table 2. Owing to the differences in range between the five datasets, the MAE was divided by the range of EEG signal to obtain the ratio. We found that the MAE ratio values obtained from the different datasets do not differ much for the EEG signals predicted by our four network models.

To further evaluate the efficacy of our predicted EEG signal in modeling real EEG signals, we considered standard deviations of the MAE ratio values. The results are listed in the MAE part of Table 2. Set A was best modeled by the small-world Kuramoto network ($10.97\pm 40.05\%$), set B by the random HR ($7.82\pm 15.90\%$) and small-world Kuramoto ($7.95\pm 15.98\%$) networks, set C by the random HR network ($7.95\pm 6.69\%$), set D by the small-world HR network ($8.17\pm 7.48\%$), and set E by the random Kuramoto network ($11.21\pm 23.89\%$). We note that the epileptic EEG signals (sets C and D, $7.95\%$ and $8.17\%$, respectively) have smaller prediction errors than the healthy EEG signals (set A, $10.97\%$) with closed eyes and comparable performance with healthy subjects with closed eyes. Data from subjects during epileptic seizure were only well modeled by the Kuramoto networks; this suggests that the epileptic brain becomes highly coherent, something captured by the Kuramoto phase oscillator network.

**Figure 6:**
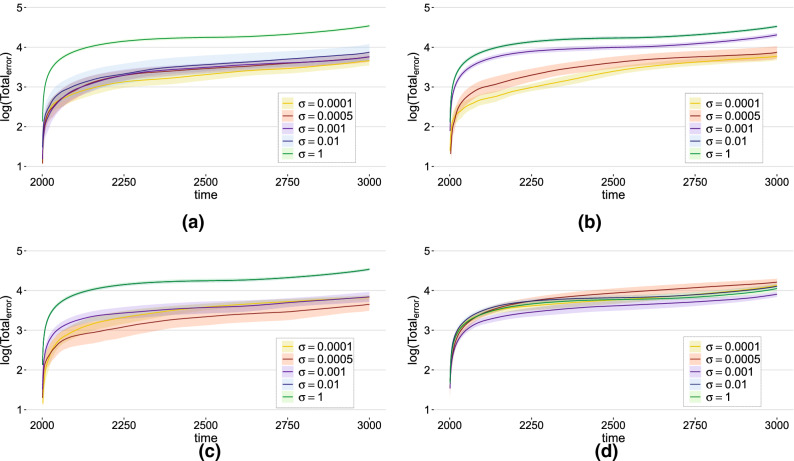
Total error of prediction from channel 4 of five subjects in dataset A. (**a**) Results from the random HR network model. (**b**) Results from the small-world HR network model. (**c**) Results from the random Kuramoto network model. (**d**) Results from the small-world Kuramoto model. The colors represent the results obtained for different coupling strength values $\sigma$.

**Table 2:** Key values for this table are the dataset (first column) and the network topologies considered: random HR (second column), small-world HR (second column), random Kuramoto (third column), and small-world Kuramoto (fourth column). There are three different sets of rows, reporting the MAE values, Hurst exponents, and power spectrum mean error, for the five datasets. Bold values represents the best result in each row.

Data	Random HR	Small-world HR	Random Kuramoto	Small-world Kuramoto
**MAE of prediction** ($\%$)				
Set A	11.28 ± 40.79	13.43 ± 58.88	12.35 ± 47.34	**10.97 ± 40.05**
Set B	**7.82 ± 15.90**	11.58 ± 50.44	9.35 ± 20.26	**7.95 ± 15.98**
Set C	**7.95 ± 6.69**	8.27 ± 9.11	9.09 ± 98.54	7.39 ± 10.49
Set D	9.40 ± 10.50	**8.17 ± 7.48**	9.91 ± 9.32	9.41 ± 12.31
Set E	45.84 ± 352.61	73.73 ± 649.34	**11.21 ± 23.89**	34.56 ± 268.48
**Hurst exponent prediction mean error**				
Set A	0.09 ± 0.06	0.19 ± 0.11	**0.08 ± 0.05**	0.17 ± 0.11
Set B	0.08 ± 0.07	**0.07 ± 0.06**	0.08 ± 0.05	0.20 ± 0.13
Set C	0.11 ± 0.07	0.13 ± 0.07	**0.08 ± 0.05**	0.16 ± 0.09
Set D	**0.06 ± 0.06**	0.09 ± 0.07	0.07 ± 0.06	0.14 ± 0.10
Set E	0.18 ± 0.10	0.15 ± 0.11	**0.10 ± 0.07**	0.17 ± 0.11
**Power spectrum prediction mean error**				
Set A	2.53 ± 1.60	2.64 ± 1.76	2.20 ± 1.37	**2.13 ± 1.65**
Set B	3.88 ± 4.57	4.30 ± 6.19	3.38 ± 4.89	**3.34 ± 4.87**
Set C	4.68 ± 3.91	4.83 ± 4.19	4.30 ± 3.42	**3.30 ± 2.44**
Set D	6.03 ± 12.84	6.14 ± 12.56	5.86 ± 11.01	**5.09 ± 15.31**
Set E	4.64 ± 5.48	5.76 ± 13.29	5.02 ± 17.35	**4.58 ± 9.67**

In addition, to ensure that our best fit models are obtained when the networks are set in the weak coupling regime responsible for the presence of the CAS phenomenon, we calculated the cumulative total error of our model as a function of time for each network model and different coupling strengths using the following formula:

2 $$\begin{aligned} Total_{error}\left( t \right) = \sum _{i=t_f+1}^{t}\left| Y_{i}-e_{i} \right| , \end{aligned}$$

which is simply the MAE multiplied by the time interval, where *Y* defines the predicted EEG signal and *e* is the actual EEG signal.

From Fig. 6, we can conclude that, independent of the types of oscillatory node dynamics and the types of network topology (excluding results from the small-world Kuramoto network), small $\sigma$ values that produce the CAS phenomenon can lead to the smaller errors between the EEG signals and the regenerated EEG signals, i.e., better prediction of the EEG signals after the weights were trained using the EEG dataset (training session). The results of datasets B, C, D, and E are shown in Supplementary Fig. A.

#### Hurst exponent

The Hurst exponent is a measure of the long-range correlation of a signal^35^, and it is broadly used to analyze EEG signals from healthy control subjects and epileptic patients^36,37^. In clinical applications, the Hurst exponent was used to identify seizure-free EEG signals from seizure interval subjects^38^ and distinguish between healthy individuals and patients suffering from epilepsy^39^.

The datasets contained healthy (A, B), seizure-free (C, D), and seizure (E) EEG signals. The Hurst exponent was calculated for all 100 single-channel EEG signals from each dataset for several sigma values. This exponent is calculated by rescaled range (R/S) analysis^40^ in a time window of 1000 time points corresponding 5.76s (Details are provided in the “Methodology” section).

We calculated the mean error and standard deviation of the difference between the Hurst exponent calculated from the predicted signal and the Hurst exponent of the experimentally obtained EEG signal. The results are listed in Table 2. In general, the random Kuramoto network model produced the smallest errors for the Hurst exponents.

#### Power spectrum

Spectral analysis is a standard method for the quantification of EEG signals^41–43^. The power spectrum reflects the frequency content of the signal or the distribution of the signal power over frequency. An important application is the measurement of event-related desynchronization (ERD)/event-related synchronization (ERS), which is widely used in brain–computer interface applications. ERD/ERS is related to the power spectrum changes at specific frequency bands during physical motor execution and mental motor imagery^15,44^.

**Figure 7:**
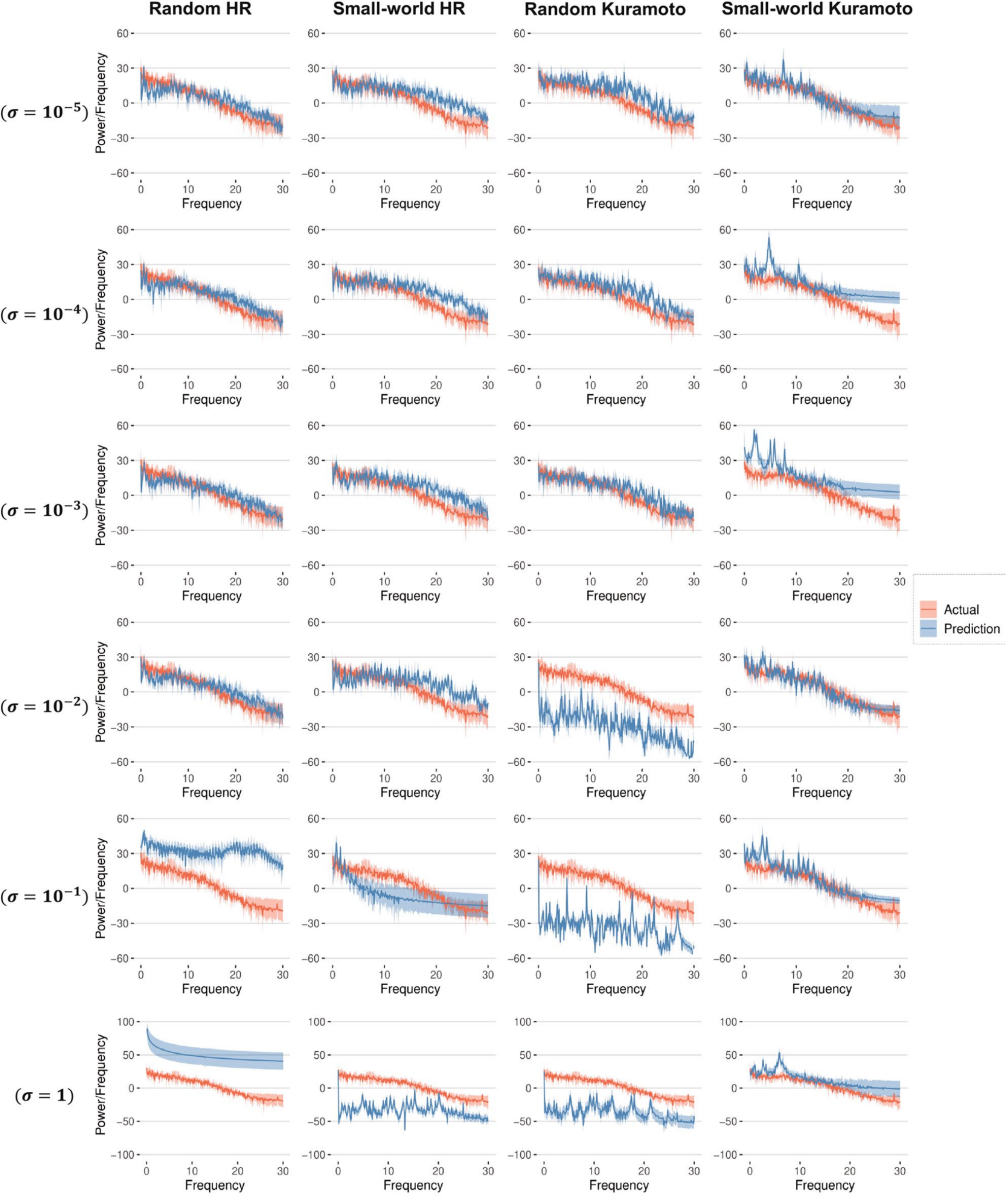
Experimental and predicted power spectrum for dataset A. The columns show the results for the random HR, small-world HR, random Kuramoto, and small-world Kuramoto models. The rows show the results for different values of coupling strength, $\sigma$.

The “actual” spectrum calculated directly from the EEG signal and the “predicted” spectrum calculated from the modeled signal in the predicting regime for the representative channel Fp1 from dataset A are presented in Fig. 7 with red and blue colors, respectively. The rows of this figure represent the power spectra (“actual” and “predicted”) for several values of $\sigma$ increasing from top to bottom. The “actual” spectra in a row are the same.

For the HR network models and the random Kuramoto network model, the difference between the predicted and original power spectra increases as $\sigma >10^{-2}$. It is worth recalling that the CAS phenomenon exists in the networks when $\sigma \leqslant 0.001$, which exactly matches the range for which the power spectrum can be well reproduced. The results of datasets B, C, D, and E are shown in Supplementary Fig. B.

## Discussion

HR neural networks operating in the CAS regime have been shown previously to reproduce EEG signals^20^, but, in this study, we reduced the fitting error of the model (Fig. 4) by using a compressed set of independent vectors produced by the PCA from the entire data generated by the network in the model instead of time series collected from randomly selected neurons. We also show that to reproduce the EEG data, one can also use dynamical networks formed by Kuramoto phase oscillators rather than coupled neuron models.

A challenge in neuroscience is to discover an oscillatory regime in which the brain functions^4^. In this study, we provide further evidence (to that provided in Ref.^20^) that the brain can operate at least locally in the CAS regime because for every type of nonlinear network studied, the best models of EEG signals are obtained with data generated from those networks that operate in the CAS regime, as shown by the results in Fig. 6. Networks in the CAS regime are characterized by small-scale clusters of neurons that are weakly coupled and behave as if they are almost synchronous neurons.

There is growing empirical support for the idea that network topology plays a crucial role in understanding brain functions. This study tests two different topological models, random and small world, for networks as well as two types of neurons, HR neurons and Kuramoto phase oscillators. Error analysis of the distance between the experimental and modeled EEG signals as well as the average differences between the feature quantities, Hurst exponent, and power spectrum are listed in Table 2. The results showed that set A was best modeled by considering the small-world Kuramoto network, set B by the random HR and small-world Kuramoto, set C by the random HR, set D by small-world HR, and set E by the random Kuramoto. We also note that the epileptic EEG signals (sets C and D) have smaller prediction errors than the healthy EEG signals (set A) with closed eyes and comparable performance with healthy subjects with closed eyes. Data from subjects during epileptic seizure were only well modeled by the Kuramoto networks; this suggests that the epileptic brain becomes highly coherent, captured by the Kuramoto phase oscillator network.

In fact, several studies reported that networks of Kuramoto phase oscillators are important for understanding seizure activity, as shown in the paper by Yan and Li^45^. These authors have inferred human brain networks from diffusion-magnetic resonance imaging in healthy controls. Thus, a computational model utilizing a delayed version of the Kuramoto model connected in a network as that inferred served as the basis for the authors postulates that frontal hubs could drive seizure activity. Another study has shown that the emergence of hypersynchrony analogous to the generation of seizures develops in a network of Kuramoto phase oscillators as a consequence of the network topology^46^.

Using other neuronal dynamical descriptions such as the Hodgkin–Huxley (HH) and the integrate-and-fire models could be considered as dynamical units of networks used for the proposed unsupervised learning approach proposed. The only requirement is that the network should be set into the CAS regime. We have considered the HH model and the networks of phase oscillators in this work because a previous work^26^ has revealed the set of parameters for which CAS exists in networks of HH and phase oscillators.

Our study used a network with $N=1000$ nodes, and we collected from each node a time series with $m=3000$ data points. Corresponding to a data frequency of 173.61 Hz, the EEG signals for 17.28 s were analyzed. This period is sufficient for classifying epilepsy seizure detection and several analyses during a short period as motor imagery classification. In the future, the data time points and the number of neurons will be increased to explore the possibility of clinical analyses. The performance of the model with other types of data such as ECG should also be studied and would depend on the type of the dynamics forming the network. Given that modified Van der Pol oscillators are a good basis for modeling ECG, they could be considered as the dynamical unit of the proposed networks.

## Conclusions

This study has shown that nonlinear networks that were set to operate in a weakly coupled regime called CAS can be used to feed data to a machine-learning-like model that can be trained by an unsupervised approach. Importantly, the output from the CAS model can reproduce EEG signals of both healthy and epileptic conditions in the predicting regime and reproduce the characteristics of the EEG signals in terms of the Hurst exponent and the power spectrum.

We have tested the performance of the CAS model based on various neuron and network types using the modeled data from healthy and epileptic subjects. Interestingly, the prediction errors between the EEG dataset and the CAS produced signals indicate that critical to better predicting the EEG signals is that artificial neurons should weakly interact with each other to ensure that the CAS can be generated. Thus, this suggests the generality of the CAS model, a weakly coupled chaotic system, in representing brain dynamics independent of the neuronal dynamics and types of the networks.

However, some limitations need to be addressed to improve this model in the future. Our model is based on a linear regression that provides a good approximation of the experimental EEG signal, but with a unique set of constant weight coefficients, a network with invariant topology, and constant coupling strength connecting the nodes. However, EEG signals are nonstationary in nature. Therefore, for long-term predictions, our model should incorporate some time-varying configurations tuned to adapt to the varying nature of the experimental signal being modeled.

Standard approaches to model EEG rely on auto-regression^11,12^ or artificial neural networks^28^. These methods, although successful in reproducing the characteristics of EEG signals, can only successfully predict the EEG signals for time intervals shorter than 1 s. The difficulty in predicting EEG signals is due to the nonstationary nature of the EEG signals. The proposed method, fundamentally based on a nonlinear network that has nodes set to operate in a CAS regime (that effectively makes their trajectories wander along a large set of periodic orbits), can lead to a successful prediction of time intervals of the order of 5.76 s.

## Methodology

### CAS phenomenon in network

Consider a network of *N* nodes, as described by

3 $$\begin{aligned} {\dot{x}}_i=\mathrm {F}_{i}\left( x_{i} \right) +\sigma \sum _{j=1}^{N}{\mathbf {K}}_{ij}\mathrm {E}\left[ \mathrm {H}\left( x_j-x_i \right) \right] , \end{aligned}$$

where $x_i\in {\mathbb {R}}^d$ is a *d*-dimensional vector describing the state variables of node *i*, $F_i$ is the *d*-dimensional vector function representing the dynamical system of node *i*, ${\mathbf {K}}_{ij}$ is the adjacent connection matrix, and *E* is the coupling function. Here, *H* is an arbitrary differential transformation. Assume in the HR model that $H(x_j)=x_j-x_i$. For the Kuramoto model, $H(x_j)=\sin (x_j-x_i)$ is a nonlinear function, which is an extension of the analysis. If the $x_{i}$ is the variable of neuron *i*, the local mean field of node *i* is defined as

4 $$\begin{aligned} {\bar{x}}_{i}\left( t \right) = \frac{1}{k_{i}}\sum _{j}{\mathbf {K}}_{ij}x_j. \end{aligned}$$

Complete synchronization appears when $x_{i}=x_{j}={\bar{x}}_i$ for all times when isolated from the network. For heterogeneity, one expects to find other weaker forms of synchronization behavior. CAS is a phenomenon that appears in a complex network that produces a weaker form of synchronization^26^. In this phenomenon, nodes are in weak interaction (weak coupling strength) and behave independently. The local cluster of neurons has roughly constant local mean fields. The CAS pattern is a solution of a simplified set of equations describing the network when ${\bar{x}}_i=C_i$. The expected value of the local mean field is defined as

5 $$\begin{aligned} C_{i}=\lim _{t\rightarrow \infty }\int {\bar{x}}_{i}\left( t \right) dt. \end{aligned}$$

The following are the two criteria for node *i* to present the CAS phenomenon:

- *Criterion 1.* The central limit theorem can be applied. Therefore, the larger the degree of a node, the smaller the variation in the local mean field.
- *Criterion 2.* The CAS pattern describes a stable periodic orbit.

In this study, the HR neurons and Kuramoto oscillator were used to model the EEG signal.

#### CAS phenomenon in HR network

The HR neuron model is a well-known model for describing the patterned activity seen in neurons. The electrical synapses can be considered as follows:

6 $$\begin{aligned} {\left\{ \begin{array}{ll} {\dot{x}}_{i}=y_{i}-ax_{i}^{3}+bx_{i}^{2}-z_{i}+\text {I}_{ext}+\sigma \sum _{j=1}^{N}{\mathbf {K}}_{ij}\mathrm {H}\left( x_{j} \right) \\ {\dot{y}}_{i}=c-dx_{i}^{2}-y_{i}\\ {\dot{z}}_{i}=-rz_{i}+sr\left( x_{i}+x_{0} \right) , \end{array}\right. } \end{aligned}$$

where $\left( x_{i},y_{i},z_{i} \right) \in {\mathbb {R}}^{3}$ are the state variables of the neuron $i,\ i=1:N$. Here, *N* is the number of neurons in the network. The parameters were selected as $a=1$, $b=3$, $c=1$, $d=5$, $s=4$, $r=0.005$, $x_0=1.618$, and $\text {I}_{ext}=3.25$, yield the HR neurons model to exhibit a multi-time-scale chaotic behavior characterized by spiking bursting. We use $\sigma$ to denote the electrical coupling strength. Simulations were performed using Matlab Simulink. The CAS patterns of node *i* are described by

7 $$\begin{aligned} {\left\{ \begin{array}{ll} {\dot{\Xi }}_{x_{i}}=\Xi _{y_{i}}-a\Xi _{x_{i}}^{3}+b\Xi _{x_{i}}^{2} -\Xi _{z_{i}}-R\Xi _{x{i}}+Q_{i}\\ {\dot{\Xi }}_{y_{i}}=c-d\Xi _{x_{i}}^{2}-\Xi _{y_{i}}\\ {\dot{\Xi }}_{z_{i}}=-r\Xi _{z_{i}}+sr\left( \Xi _{x_{i}}+x_{0} \right) , \end{array}\right. } \end{aligned}$$

where $R_{i}= p_{i}$, $Q_{i}=p_{i}C_{i}$, $p_{i}={\sigma }k_{i}$ and $C_{i}\approx \left( 1/k_{i} \right) \sum _{j=1}^{N}{{\mathbf {K}}_{ij}x_{j}}$. To illustrate the presence of the CAS phenomenon, we considered a random network formed by $N=1000$ neurons.

#### CAS phenomenon in Kuramoto oscillators

The Kuramoto model was used to simulate brain interactions through synchrony on the basis of structural and functional implications of the organization of brain connectivity^47^. The dynamics of node *i* are described by

8 $$\begin{aligned} {\dot{\theta }}_{i}=\omega _{i}+p_{i}{\tilde{r}}_{i}\sin \left( {\bar{\theta }} _{i} -\theta _{i} \right) , \end{aligned}$$

where $p=0.01$ is the probability that each two nodes are connected and $\omega _{i}$ is the natural frequency of node *i* selected randomly from $\left[ -\pi , \pi \right]$. Here, ${\tilde{r}}_{i}$ is the coupling strength of node *i*. The CAS patterns of node *i* are described by

9 $$\begin{aligned} {\dot{\Xi }}_{i}=\omega _{i}+p_{i}{\tilde{r}}_{i}\sin \left( C_{i}-\Xi _{i}\right) . \end{aligned}$$

Equation (9) describes a periodic orbit regardless of the values of parameters $\omega , p_{i}$ and ${\tilde{r}}$ because it is an autonomous two-dimensional system; chaos cannot exist^26^. Therefore, **criterion 2** is always satisfied in Kuramoto oscillators.

#### Simulation of the neuronal networks to predict a given series of EEG signals

As an overall flow, HR neurons or Kuramoto oscillators were implemented in each node, and random or small-world networks were generated for neuronal networks. To test this hypothesis, we verified the types of neurons and network structures in the predicting regime (Fig. 1). The connectivity matrix ${\mathbf {K}}$ defines the weightings of the synaptic connections between neurons, defined by the electrical coupling strength $\sigma$. The neuron networks are obtained with $\sigma$ in the range from 0 to 1.2. The connections ${\mathbf {K}}$ are generated with random and small-world 1000-node networks. The median node degree is 10. A total of 3000 neurons were simulated using the Brain Dynamics Toolbox^48^ for HR neurons and Kuramoto oscillators. Then, the local mean field *C* of each node is calculated using Eq. (4), and *C* are plugged into the differential equation to obtain the CAS pattern. For the HR model, we used $X=\left\{ x_i \right\} _{i\in 1:1000}$ as a matrix composed of membrane potentials of the simulated neurons. For the Kuramoto model, we used the matrix $X=\left\{ \theta _i \right\} _{i\in 1:1000}$ as a combination of neuron oscillations. Finally, using the matrix $\mathbf{X }(t)$ defined in Eq. (10), each $3000\times 1000$ neuron network is reduced by using the PCA method. The dimensionally reduced matrix maintains $99\%$ of the information of the original matrix. Training datasets of the EEG signals were used to determine the weight values of the individual neurons to fit the EEG signals as a function of time.

### CAS-network-based model for EEG signals

To model the EEG signals, we used the property of linear algebra. Given an unknown vector $a\in {\mathbb {R}}^{n \times 1}$ of trained coefficients, a known matrix $\mathbf{X } \in {\mathbb {R}}^{m \times n}$ obtained using the methods to be further explained but are a function of measurements obtained from the dynamical network (where *m* denotes the number of measurements obtained or the discrete time interval), and a known vector $b\in {\mathbb {R}}^{m \times 1}$ (which is set to be equal to an EEG signal), the following equation

10 $$\begin{aligned} {\varvec{X}} a = b, \end{aligned}$$

has a unique solution by using least square method^49^

11 $$\begin{aligned} a={\varvec{X}}^{+} b. \end{aligned}$$

Here, ${\varvec{X}}^{+} \in {\mathbb {R}}^{n \times m}$ is the Moore–Penrose pseudoinverse of matrix ${\varvec{X}}$.

Given a training set of data from the EEG signals, denoted by $b_0$, we calculated the trained coefficients $a_0$ using

12 $$\begin{aligned} a_0={\varvec{X}}^{+} b_0. \end{aligned}$$

Our CAS-network-based model for the EEG whose training set is $b_0$ is thus expressed as follows:

13 $$\begin{aligned} Y = {\varvec{X}} a_0, \end{aligned}$$

where $Y \in {\mathbb {R}}^{(t_m-t_1) \times 1}$ is our EEG model for a time interval of $t_m-t_1$, $X \in {\mathbb {R}}^{(t_m-t_1) \times n}$ is a matrix constructed from the dynamical network by taking $(t_m - t_1)$ observations, and $a_0$ the vector of coefficients trained by Eq. (12).

To validate our model, we calculated the MAE function, which measures the averaged difference between the modeled EEG signal and the actual EEG signal denoted by $e \in {\mathbb {R}}^{(t_m-t_1) \times 1}$:

14 $$\begin{aligned} MAE=\frac{\sum _{t=t_{f}+1}^{t_{m}}\left| e_t - Y_t \right| }{(t_m - t_f)}. \end{aligned}$$

### Dimension reduction of ${\varvec{X}}^* \in {\mathbb {R}}^{m \times N}$ by PCA

Define ${\varvec{X}}^* \in {\mathbb {R}}^{m \times N}$ as the matrix that contains full information about the dynamical network operating in the CAS regime. Every row is a time series of values obtained from a node of the network, and the entire network is set with a total of *N* nodes.

The matrix $X^{*}$ can be factorized using SVD

15 $$\begin{aligned} {\varvec{X}}^{*} = U \Sigma V^{T}, \end{aligned}$$

where $K \le \{m,N\}$ is the rank of matrix ${\varvec{X}}^*$ and $\sigma _{1}\ge \sigma _{2}\ge \dots \ge \sigma _{K}$, with $\sigma _i = \Sigma _{ii}$ are the singular values of ${\varvec{X}}^*$. Here, $\Sigma \in {\mathbb {R}}^{m \times N}$. $U \in {\mathbb {R}}^{m \times m}$ is the left singular vector, and $V \in {\mathbb {R}}^{N \times N}$ is the right singular vector of ${\varvec{X}}^*$.

The eigenvector with the highest eigenvalue is the principal component of ${\varvec{X}}^*$. In fact, the eigenvector with the largest eigenvalue represents the most significant relationship between the dimensions. An approximate compact matrix can be constructed with a specific rank *k* such that $k<K$, whose singular values only contain the *k* largest singular values of ${\varvec{X}}^*$. Using this approach, the matrix ${\varvec{X}}^*$ can be approximated by

16 $$\begin{aligned} {\varvec{X}}^* \approx {\varvec{X}}_{k}=U_{k}\Sigma _{k}\left( V_{k}\right) ^{T}. \end{aligned}$$

Matrix $U_k \in {\mathbb {R}}^{m \times k}$, $\Sigma _{k} \in {\mathbb {R}}^{k \times k}$, and $V_k^{T} \in {\mathbb {R}}^{k\times N}$. Making the definition

17 $$\begin{aligned} U = (u_1\ u_2\ u_3 \ldots u_k) \text{ and } V = (v_1\ v_2\ v_3\ \ldots \ v_k), \end{aligned}$$

we can write that

18 $$\begin{aligned} {\varvec{X}}_{k} = \sum _i^k \sigma _i u_i v_i^T. \end{aligned}$$

The standard measure of the quality of $X_{k}$ is the proportion of total variance, which is defined by the Frobenius norm of the difference between two matrices:

19 $$\begin{aligned} \frac{\left\| {\varvec{X}}_{k} \right\| _F^{2}}{\left\| {\varvec{X}}^{*} \right\| _F^2}=\frac{\sum _{i=1}^{k}\sigma _{i}^2}{\sum _{i=1}^{K}\sigma _{i}^2}. \end{aligned}$$

Thus, the proportion of the total variance is higher if *k* is larger. This is an important theorem that helps determine the matrix approximation based on the amount of information required. Therefore, we want to maintain at least $99\%$ of the information of ${\varvec{X}}^{*}$ and selected the smallest *k* such that

20 $$\begin{aligned} \frac{\sum _{i=1}^{k}\sigma _{i}^2}{\sum _{i=1}^{K}\sigma _{i}^{2}} = 99\%. \end{aligned}$$

Suppose that *n* is the value of *k* such that the proportion of total variance is equal to $99\%$. The truncated $m \times n$ of matrix ${\varvec{X}}$ can be obtained by considering only the first *n* largest singular values and their singular vectors^50,51^:

21 $$\begin{aligned} {\varvec{X}}_{m\times n}=U_{m\times n}*\Sigma _{n \times n}={\varvec{X}}^{*}*V_{N \times n}, \end{aligned}$$

where $V_{N\times n}$ is the *n* first columns of *V*. These *n* vectors in ${\varvec{X}}_{m\times n}$ are called the principal components that are linearly uncorrelated and have $99\%$ variance with ${\varvec{X}}^{*}$.

### Hurst exponent

Let a single EEG signal to be represented by $e\in {\mathbb {R}}^{\left( t_{m}-t_{f}\right) \times 1}$, with $e=\{e_1, e_2, \ldots , e_n\}$, with $n=t_m-t_f=1000$, which is the time interval considered in our study. The average value of *e* is denoted by ${\mathbb {E}}\left( e \right)$.

Defining the adjusted range as

22 $$\begin{aligned} R(n)= max \left( 0,w_1,w_2,\ldots ,w_n \right) - min \left( 0,w_1,w_2,\ldots ,w_n \right) , \end{aligned}$$

where for each $k\in [1:n],\ w_k=\sum _{j=1}^{k}\left( e_j-k{\mathbb {E}}\left( e \right) \right)$, then the Hurst exponent is defined by finding the scaling that fits to

23 $$\begin{aligned} \frac{R(n)}{S(n)}\sim cn^H \end{aligned}$$

with *S*(*n*) representing the standard deviation of *e*. An estimation of the Hurst exponent adopted in this work can be calculated by using a rescaled range formula^35^: $\frac{R(n)}{S(n)}\sim \left( 2^{(2H-1)}-1 \right) n^H$.

## Supplementary information

Supplementary Figures.

## Data Availability

The datasets generated during the current study are available on request from the corresponding author.
