# Supplementary material for: Collective almost synchronization-based model to extract and predict features of EEG signals
Source: Sci Rep. 2020 Oct 1;10:16342. doi: 10.1038/s41598-020-73346-z (PMC7530765; doi:10.1038/s41598-020-73346-z)
Supplement: Supplementary file 1 — Supplementary Figures. [file 41598_2020_73346_MOESM1_ESM.pdf]

Supplementary materials for: Collective Almost Synchronization-based model to extract and predict features of EEG signals

Phuong Thi Mai Nguyen[1], **Yoshikatsu Hayashi**[2], **Murilo Da Silva Baptista**[3], **and Toshiyuki Kondo**[1,*]

[1]Department of Computer and Information Sciences, Tokyo University of Agriculture and Technology, Tokyo, 184-8588, Japan.
[2]Biomedical Science/Engineering, School of Biological Sciences, University of Reading, Reading, RG6 6UR, United Kingdom.
[3]Institute for Complex System and Mathematical Biology, University of Aberdeen AB24 3UE, United Kingdom.
[*]Corresponding author: t_kondo@cc.tuat.ac.jp

Supplementary Figures

Supplementary Figure A

Figures 1, 2, 3, and 4 show the total errors between EEG signals and generated EEG signals for each network model and different coupling strengths. Small σ values that produce CAS can lead to the smaller errors i.e., better prediction of the EEG signals after the training session.

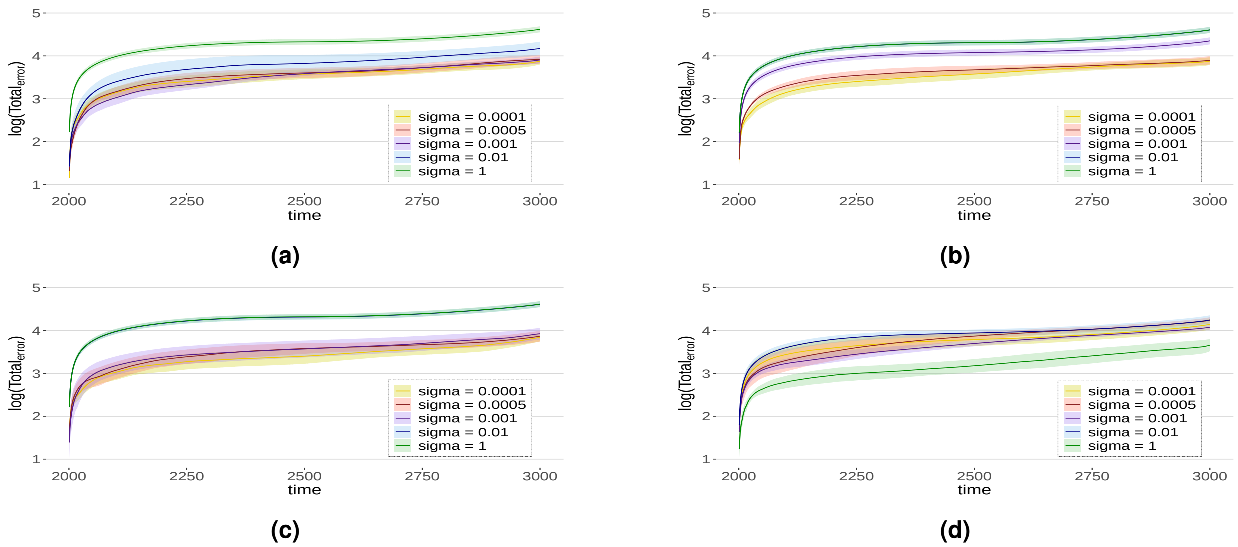

Supplementary Figure 1. Total error of prediction from channel 4 of five subjects in dataset B. (**a**) Results from the random HR network model. (**b**) Results from the small-world HR network model. (**c**) Results from the random Kuramoto network model. (**d**) Results from the small-world Kuramoto model. The colors represent the results obtained for different coupling strength values σ.

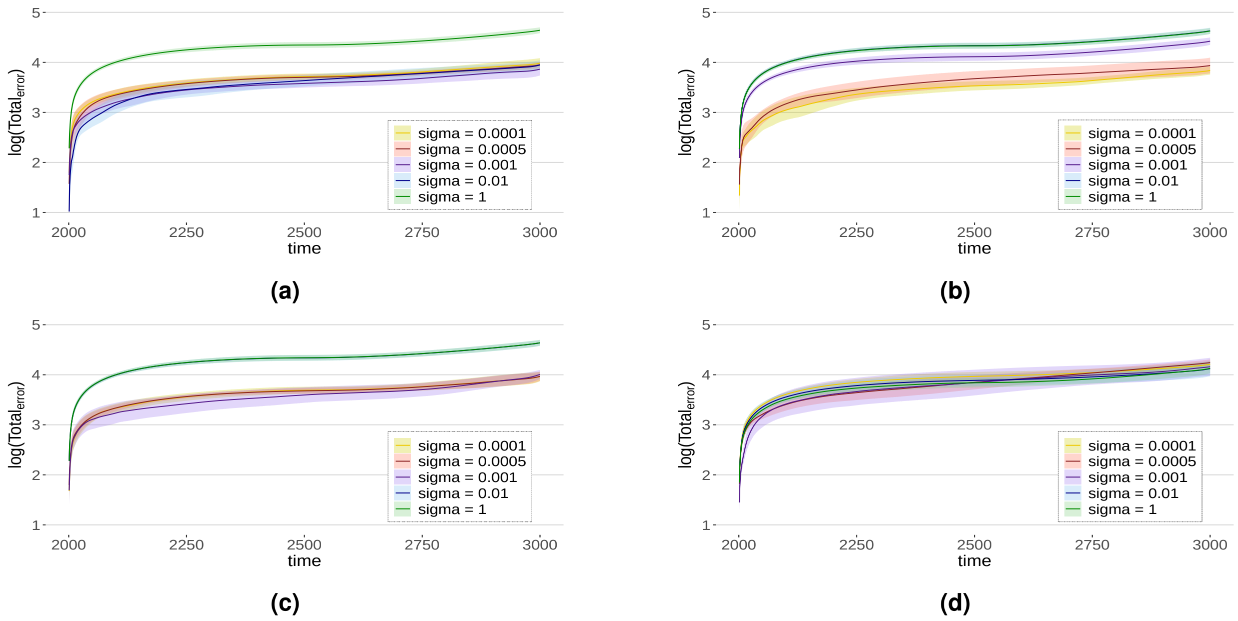

Supplementary Figure 2. Total error of prediction from channel 4 of five subjects in dataset C. (**a**) Results from the random HR network model. (**b**) Results from the small-world HR network model. (**c**) Results from the random Kuramoto network model. (**d**) Results from the small-world Kuramoto model. The colors represent the results obtained for different coupling strength values σ.

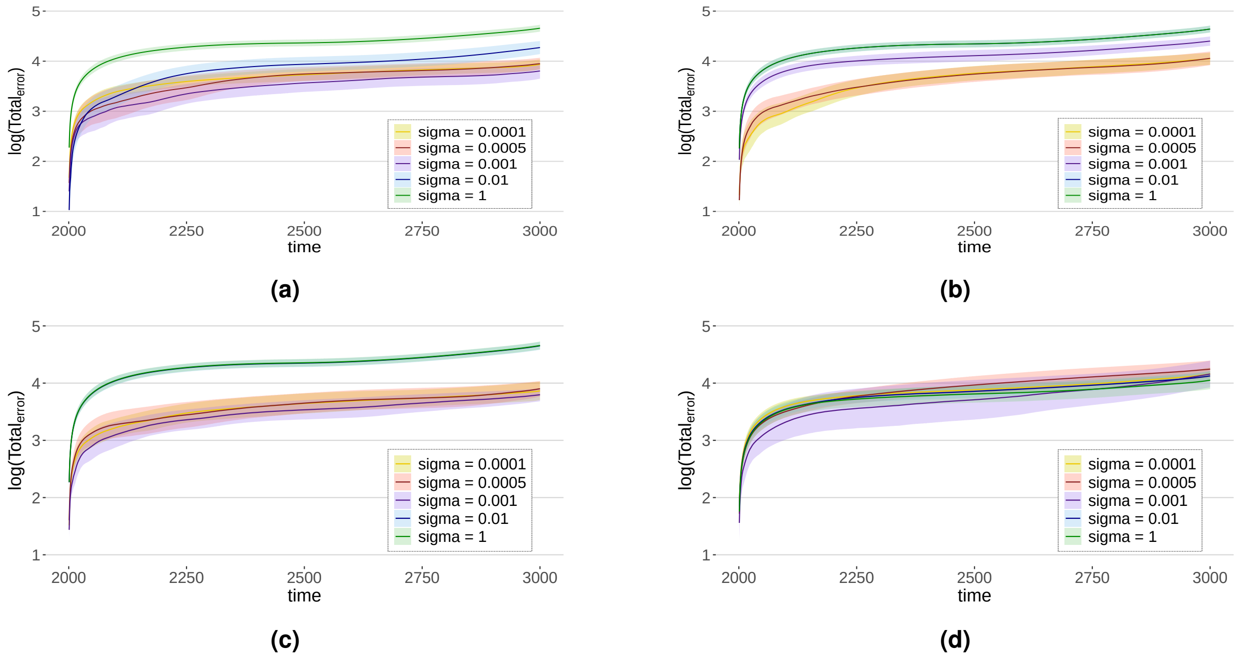

Supplementary Figure 3. Total error of prediction from channel 4 of five subjects in dataset D. (**a**) Results from the random HR network model. (**b**) Results from the small-world HR network model. (**c**) Results from the random Kuramoto network model. (**d**) Results from the small-world Kuramoto model. The colors represent the results obtained for different coupling strength values σ.

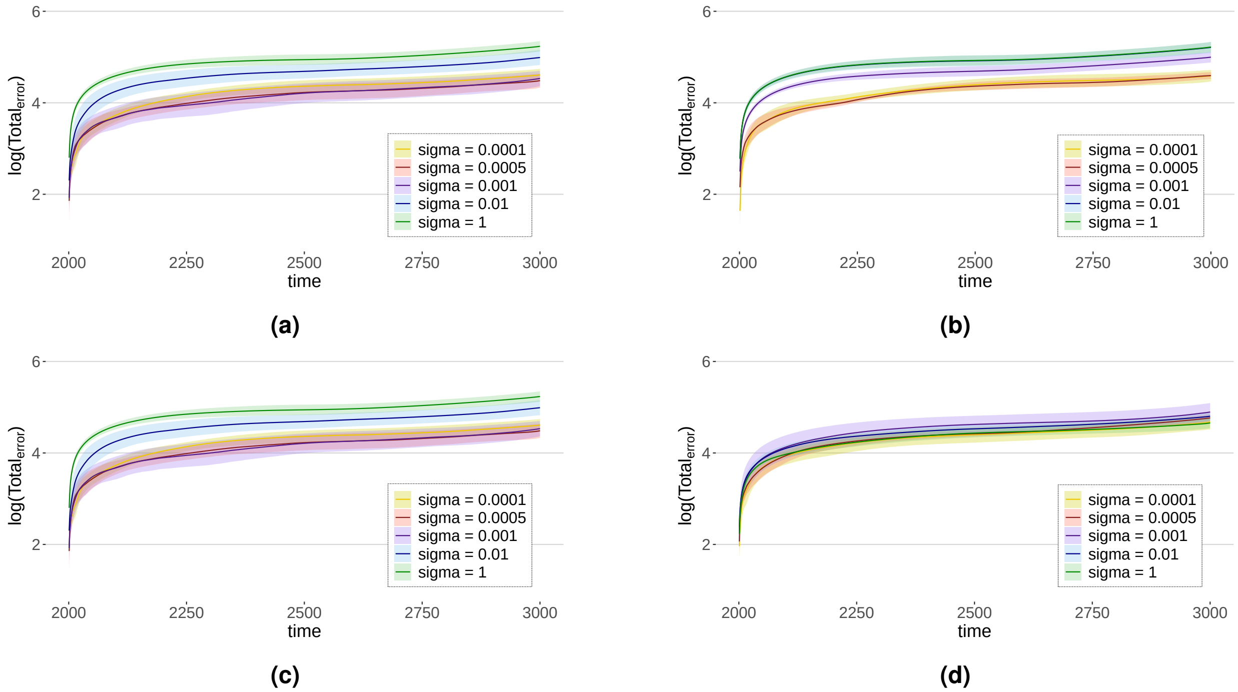

Supplementary Figure 4. Total error of prediction from channel 4 of five subjects in dataset E. (**a**) Results from the random HR network model. (**b**) Results from the small-world HR network model. (**c**) Results from the random Kuramoto network model. (**d**) Results from the small-world Kuramoto model. The colors represent the results obtained for different coupling strength values σ.

Supplementary Figure B

Figures 5, 6, 7, and 8 show the spectrum calculated directly from the EEG signals and the predicted EEG signals of channel Fp1 for each network model and different coupling strengths. The rows of this figure represent the power spectra ("actual" and "prediction") for several values of σ increased from up to down.

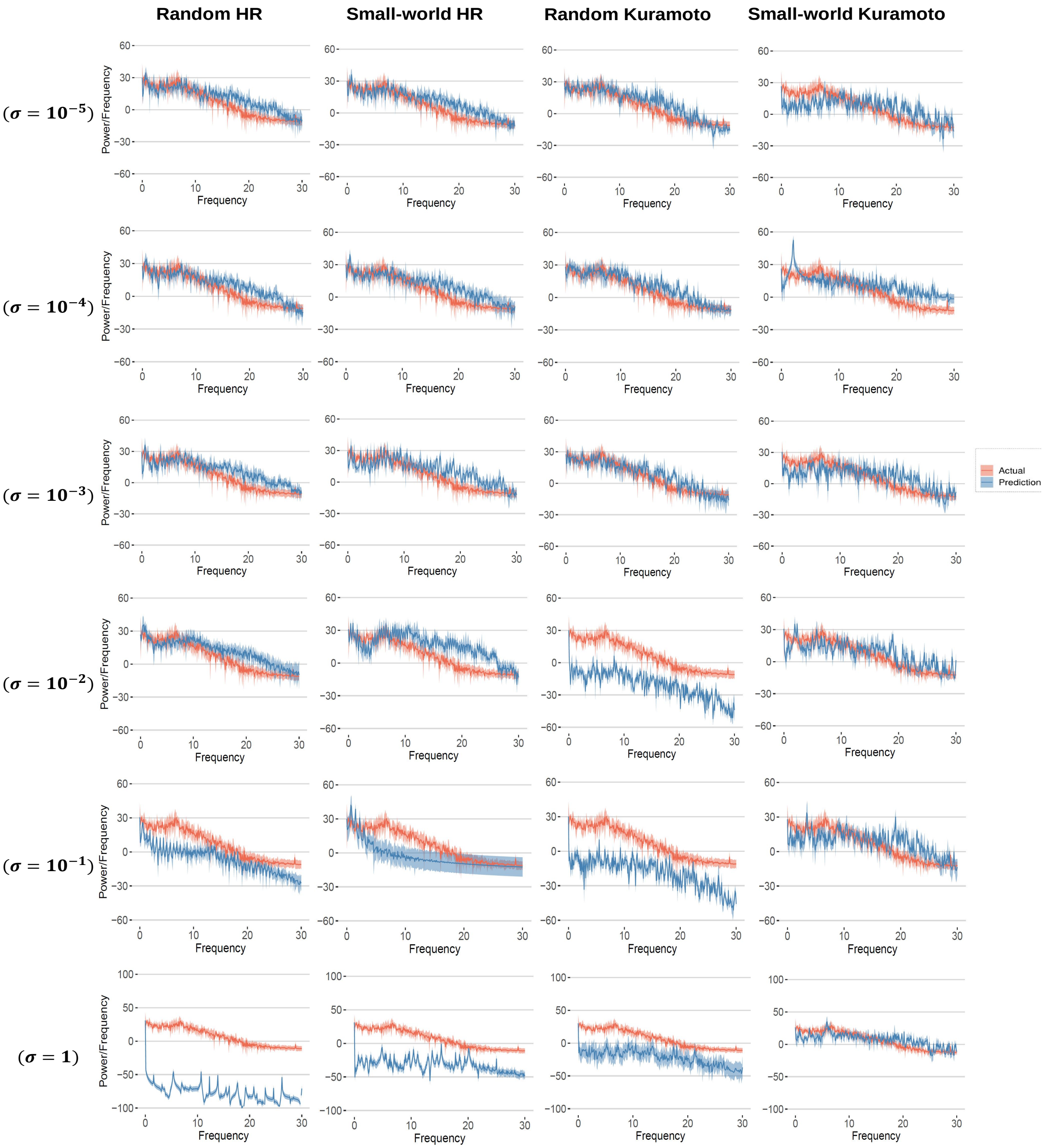

Supplementary Figure 5. Experimental and predicted power spectrum for dataset B. The columns show the results for the random HR, small-world HR, random Kuramoto, and small-world Kuramoto models. The rows show the results for different values of coupling strength, σ.

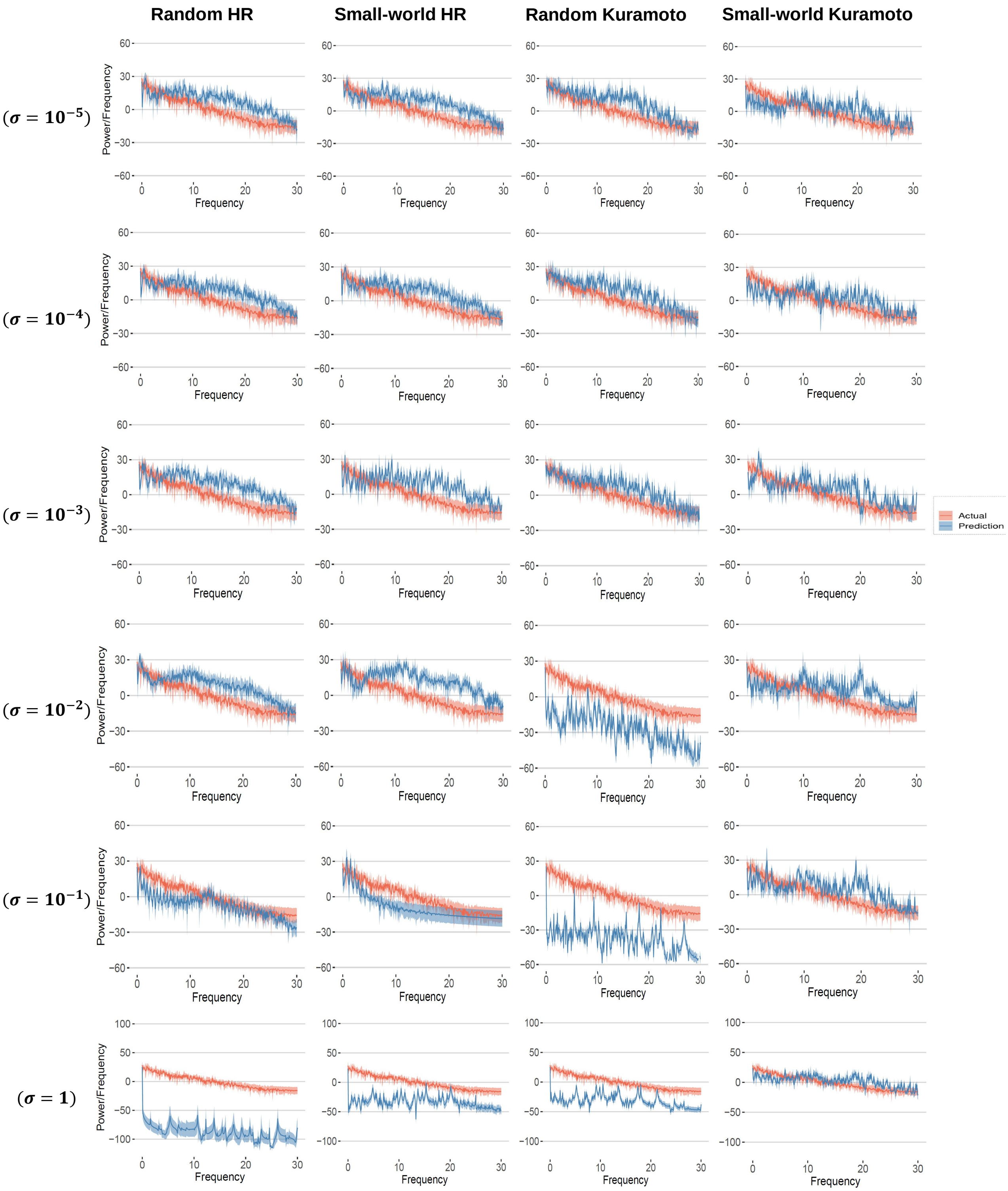

Supplementary Figure 6. **Experimental and predicted power spectrum for dataset C**. The columns show the results for the random HR, small-world HR, random Kuramoto, and small-world Kuramoto models. The rows show the results for different values of coupling strength, σ.

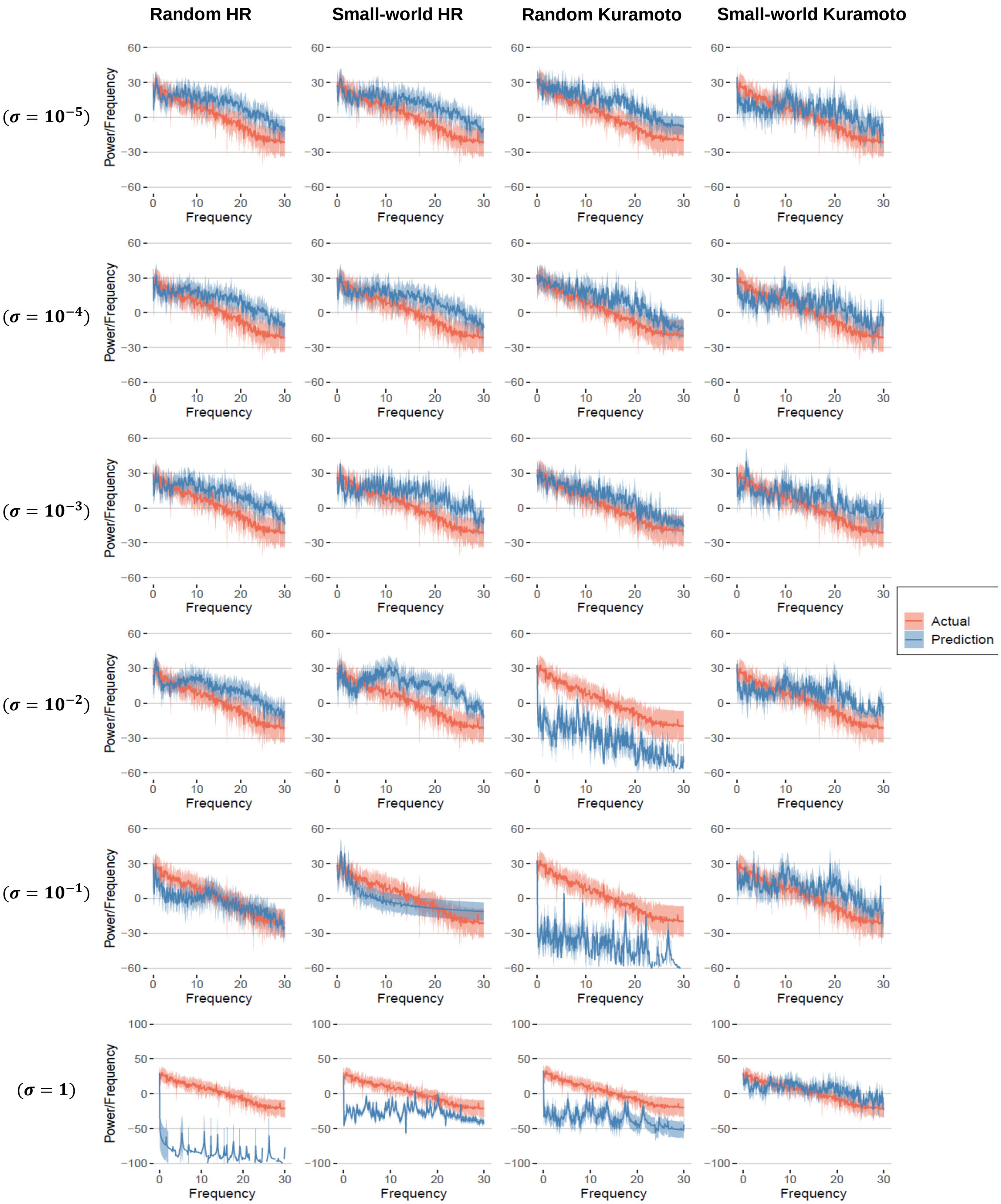

Supplementary Figure 7. Experimental and predicted power spectrum for dataset D. The columns show the results for the random HR, small-world HR, random Kuramoto, and small-world Kuramoto models. The rows show the results for different values of coupling strength, σ.

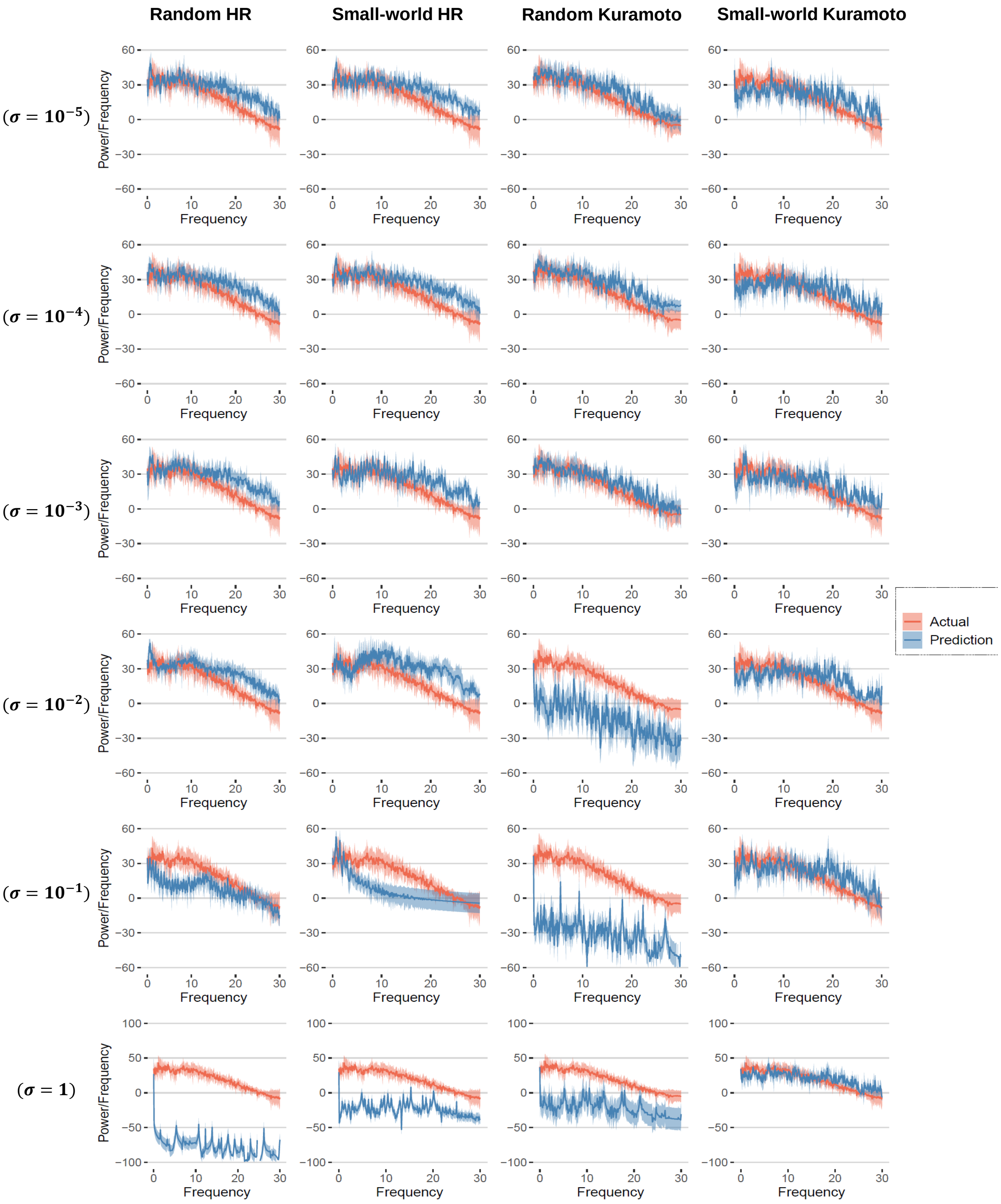

Supplementary Figure 8. Experimental and predicted power spectrum for dataset E. The columns show the results for the random HR, small-world HR, random Kuramoto, and small-world Kuramoto models. The rows show the results for different values of coupling strength, σ.
